# Low *Trypanosoma cruzi* transmission risk to humans in the Trans-Pecos region of Texas

**DOI:** 10.1016/j.parepi.2020.e00180

**Published:** 2020-09-09

**Authors:** Kyndall C. Dye-Braumuller, M. Katherine Lynn, Rodion Gorchakov, Sarah M. Gunter, Rebecca M. Berry, Kristy O. Murray, Melissa S. Nolan

**Affiliations:** a University of South Carolina, Columbia, SC, USA; b Harris County Public Health, Houston, TX, USA; c Baylor College of Medicine and Texas Children's Hospital, Houston, TX, USA

**Keywords:** *Trypanosoma cruzi*, *Triatoma rubida*, *Triatoma gerstaeckeri*, *Triatoma protracta*, *Pimeliaphilus*, Mites, Ectoparasites, Texas, Chagas disease, Human infection

## Abstract

In the Trans-Pecos region of Texas, reports of domestic triatomine bites were common (67%), with 36% of residentially collected triatomines positive for *Trypanosoma cruzi*. Despite the transmission potential, no human infections were detected. Collected *Triatoma rubida* species were themselves frequently parasitized with mites.

## 1 Introduction

Triatomines are competent vectors of *Trypanosoma cruzi*, the etiological agent for Chagas disease (CD) (Garcia et al., 2015). Southwestern states account for the highest triatomine density and species diversity in the US (Bern et al., 2011). Reports documenting *Triatoma gerstaeckeri*, *T. protracta*, and *T. rubida* in the Trans-Pecos region of Texas date back to the 1930s (Garcia et al., 2015; Bern et al., 2011; Wood, 1941). CD is a parasitic infection that leads to irreversible heart damage and death in 20–30% of human cases (Bern et al., 2011). Inadequate surveillance and physician knowledge make it difficult to determine the true burden of autochthonous infection in the US, despite consistent reports of domestic invasion and anaphylaxis resulting from triatomine bites in the southwest (Behrens-Bradley et al., 2019; Garcia et al., 2015; Stimpert and Montgomery, 2010; Klotz et al., 2016). This investigation focuses on an area in Texas with a long history of human bites, unexplored transmission dynamics, and unknown human infection prevalence (Garcia et al., 2015).

## 2 Materials and methods

We tested residents of Big Bend National Park (BBNP) and Terlingua, TX for *Trypanosoma cruzi* infection due to reported high rates of canine infection and reported domestic exposure to triatomines. This project was reviewed and approved by the Institutional Review Board at BCM and the BBNP Research Permit Board. All residents were offered point-of-care testing via Chagas STAT-PAK (Chembio Diagnostic Systems, Inc., Medford, NY), and secondary serologic testing via Hemagen Chagas Kit (Hemagen Diagnostics Inc., Columbia, MD). Any suspected positives were confirmed using Chagatest ELISA recombinante v3.0 (Wiener Lab, Argentina).

Residential assessments in the form of a residential exposure survey were conducted at participants' homes which assessed housing conditions (type and integrity of structures), environmental and socio-economic factors, and vector exposure (history of bug bites or kissing bug-specific reports). Concurrently, we conducted home surveys for potential triatomine habitats such as suspected rodent burrows or debris piles.

Triatomines were collected from resident homes by study participants, our team, and overnight traps. Trap specifications have been previously described (Dye-Braumuller et al., 2019). Collections in BBNP were conducted nightly through UV blacklight (BioQuip Products, Rancho Dominguez, CA) trapping and active searching. All triatomines collected were stored at −80 °C and morphologically identified to species and sex using a dichotomous key (Lent and Wygodzinsky, 1979). Posterior abdominal segments 2–3 were exsected for *Trypanosoma cruzi* detection; DNA was extracted using Quick-DNA Tissue/Insect Miniprep Kits (Zymo Research, Irvine, CA). PCR was used for triatomine species confirmation through sequencing the 16S rRNA gene. Isolates of *Trypanosoma cruzi* positive insects underwent qPCR and phylogenetic analysis to identify discrete typing unit (DTU) (Garcia et al., 2017).

## 3 Results and discussion

Between March and May 2017, 85 residents participated in diagnostic testing with 81 providing a serologic sample for secondary testing. Of the 85 initially screened, 2 were positive on Chagas STAT-PAK. All 81 participants who permitted secondary serologic testing were negative including the two who were initially positive by STAT-PAK, suggesting they were false positives. Of the 85 participants, 35 lived within BBNP boundaries, at a housing community near Panther Junction Visitor Center. The other 50 lived in neighboring auspices of Terlingua, TX. Participants were mostly non-Hispanic Caucasian (88%), middle-aged (average age of 55 yrs), and equally represented genders (49% female).

The majority of participants (65%) completed a residential exposure survey. The majority lived in single-family homes (84%), with limited mobility (average 12 years at current residence). Most achieved undergraduate (45%) or graduate degrees (33%) and earned annual household incomes between $50 k–100 k (41%). Peridomestic and sylvatic human exposures were common. Most residents reported a “bug pest” issue (84%), with pest issues inside the home (69%) frequently reported. Most reported unexplained insect bites while inside (84%), including bites while sleeping (76%). Participants specifically noted (67%) a triatomine bite history averaging >15 years (range 1–37 years). Insect bite reactions were mostly inflammatory; however, some recounted systemic itching and hives, with some (22%) describing progressively worsening reactions with each subsequent triatomine bite. Evidence of potential triatomine habitats adjacent to residences were common, including small animal nests (53%) and woodpiles (62%). Many respondents (69%) had backyards bordering forest edge or bushland with 36 (65%) living on >5 acres of land.

**Fig. 1:**
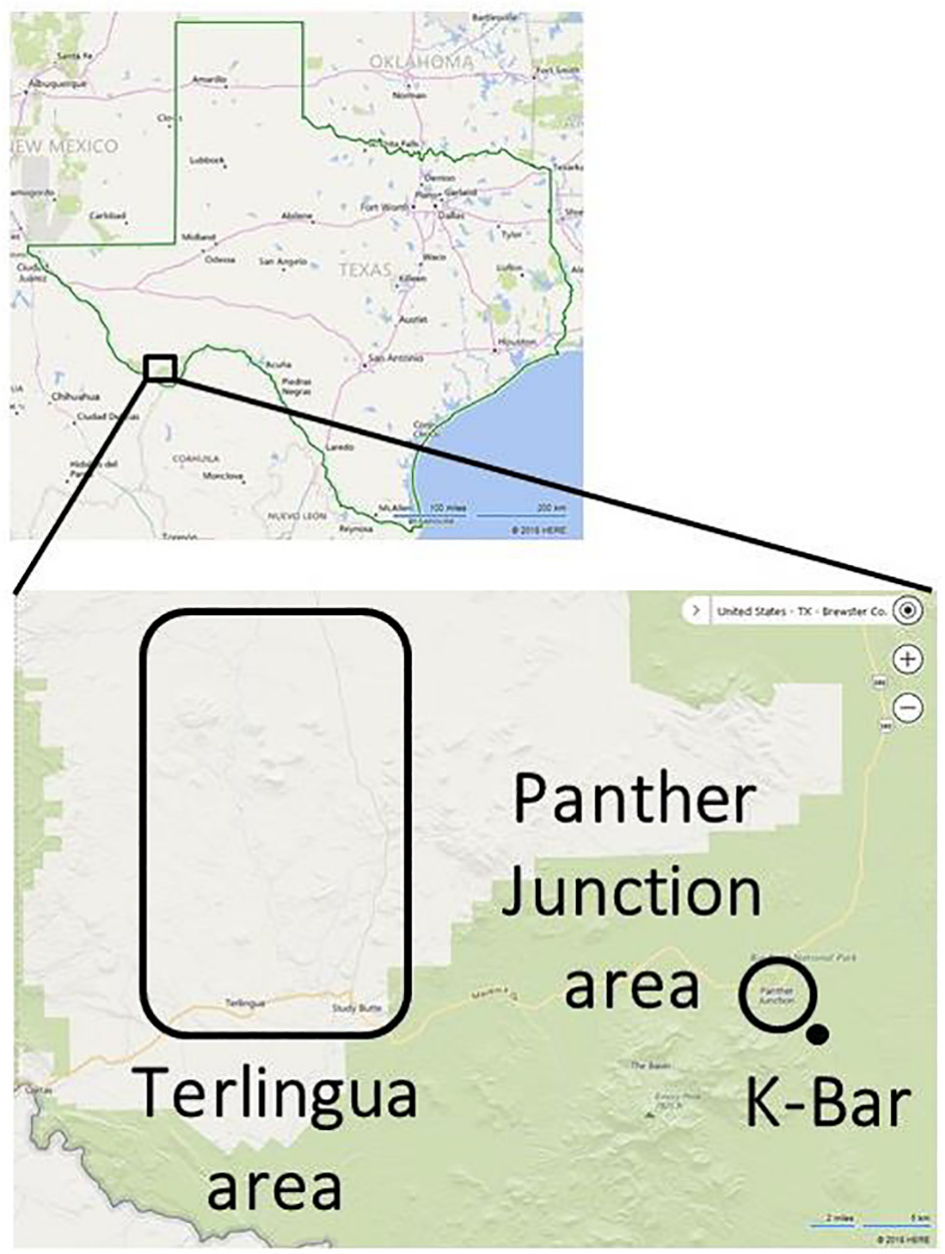
Geographic locations of human resident surveillance and triatomine collections.

A total 88 triatomines were collected at one of three sites: within the Terlingua city area or within BBNP at either the K-bar cabin or in Panther Junction (Table 1 **and** Fig. 1). Most were collected at K-Bar cabin-BBNP (*N* = 41), followed by Terlingua (*N* = 33) and Panther Junction-BBNP residences (*N* = 14). Triatomines were collected by active inspection of potential habitats (32%), trapping by UV light or baited cardboard box (22%) or through passive collection from residents (47%). Of the trapping methods, the majority originated from UV light methods. All triatomines from Terlingua were identified as *T. rubida* and were *Trypanosoma cruzi* negative. In Panther Junction-BBNP, both *T. rubida* and *T. protracta* were collected, with 29% positive for *Trypanosoma cruzi*, all identified as *T. rubida.* Finally, at K-Bar cabin-BBNP, *T. rubida* dominated collections with one *T. gerstaeckeri*. Including this *T. gerstaeckeri*, 68% of triatomines were positive for *Trypanosoma cruzi*. Overall, 36% of 88 triatomines collected from all three study sites were positive for *Trypanosoma cruzi*, all having TcI DTU.

**Table 1:** Triatomine insect analysis.

Triatomine Collection Location	No. collected	Collection method, No. (% of collected)	Lifestage, No. (% of collected)	*Triatoma* species, No. (% of collected)	No. *Trypanosoma cruzi* positive (% of collected)	*Trypanosoma cruzi* DTU, No. (% of positive)
Terlingua	**33**	Active, **6** (18) Passive, **27** (82)	Adult, **9** (27) Nymph, **24** (73)	*T. rubida*, **33** (100)	**0** (0) **0** (0)	N/A
Panther Junction	**14**	Passive, **14** (100)	Adult, **14** (100)	*T. rubida*, **12** (86) *T. protracta*, **2** (14)	**4** (29) **0** (0)	TcI, **4** (100)
K-Bar cabin	**41**	Active, **22** (54) Trapping, **19** (46)	Adult, **41** (100)	*T. rubida*, **40** (98) *T. gerstaeckeri*, **1** (2)	**27** (66) **1** (2)	TcI, **28** (100)
Total	**88**	Active, **28** (32) Passive, **41** (47) Trapping, **19** (22)	Adult, **64** (73) Nymph, **24** (27)	*T. rubida*, **85** (97) *T. protracta*, **2** (2) *T. gerstaeckeri*, **1** (1)	**32** (36)	TcI, **32** (100)

Triatomines from the three study sites were analyzed and identified from collection method, life stage, species, and *Trypanosoma cruzi* infection.

While identifying triatomine species morphologically using a microscope, we noted several specimens parasitized by small, orange-colored mites, Table 2. Roughly 25% of all collected *T. rubida* were parasitized. Average number of mites per triatomine was 1.8, however, male *T. rubida* had slightly higher average mite load (2.2 per triatomine) than females (1.3). All mites were ventrally located on the triatomine integument; parasitism by body part order followed legs > thorax > abdomen > head.

**Table 2:** Mite presence on collected *Triatoma rubida* (*N* = 21).

Total No. *T. rubida* with mites	Average No. mites / *T. rubida*	Average No. mites / F *T. rubida*	Average No. mites / M *T. rubida*	Total mite distribution			
				Head	Thorax	Legs	Abdomen
21	1.8	1.3	2.2	1	13	18	6

Parasitic mites were found on triatomines; parasitization location on the triatomine integument was documented.

This investigation evaluated CD transmission risk in an area with a long-documented history of triatomine exposure. We did not identify any human infections; however, two-thirds of residents reported known triatomine bites, and one-third of triatomines collected from residences were *Trypanosoma cruzi* positive. Documented sylvatic *Trypanosoma cruzi* transmission in the area is established, with moderate (<40%) infection rates in triatomines and in mammalian reservoirs (Wood, 1941; Ikenga and Richerson, 1984; Kjos et al., 2009; Curtis-Robles et al., 2018)**.** On average, participants had a 15-year history of recurring triatomine bites, and all collected insects were found in or around homes. Despite substantial infection in triatomines and mammalian reservoirs, results of this study suggest individuals in this area of Texas are not at high risk for contracting CD. Similar results were identified in southern Arizona, a comparable desert climate with great insect exposure but low levels of human infection (Behrens-Bradley et al., 2019).

This is the first published report of ectoparasitic mites on triatomines from this region of Texas (Martí et al., 2017). These findings are consistent with additional trapping along the Arizona-Mexico border (M. Nolan, unpublished data). Eight species of ectoparasitic mites have been reported in North and South America since 1944; four found on US triatomines: *Pimeliaphilus andersoni*, *P. sanguisugae*, *P. plumifer*, and *P. calimesae (*Martí et al., *2017**)*. Since *P. plumifer* is the only mite that has been found on *T. rubida*, this is most likely the species found in our study. Our study revealed slightly higher *Pimeliaphilus* infestation rates than previous reports (Anderson, 1968; Martinez-Sanchez et al., 2007).

*Pimeliaphilus* mites can significantly reduce fitness of triatomine hosts through reduced egg viability and increased mortality (Anderson, 1968; Martinez-Sanchez et al., 2007). There is no evidence that these parasitic mites ingest *Trypanosoma cruzi* from triatomines as the trypanosome does not enter triatomine hemolymph, however they have ingested and may transmit *Trypanosoma rangeli* between triatomine species (Anderson, 1968). It is possible that these parasitic mites can reduce fitness of triatomines to a level that may reduce transmission risk to humans. Further studies should assess parasitism rates in sylvatic versus residential species, identification of blood meal sources and serological markers of triatomine exposures.

## 4 Conclusions

This study highlights the nearly 40 years of high risk exposure to known *Trypanosoma cruzi-*infected triatomines from the Trans-Pecos region, along with a lack of CD transmission to humans. Results are consistent with previous data in an analogous ecological setting in Arizona (Behrens-Bradley et al., 2019). Triatomine transmission of *Trypanosoma cruzi* to humans depends on post bloodmeal defecation behavior, number of human exposures, and vector parasite load (Klotz et al., 2016). Evidence points to longer intervals between bloodmeal and elimination in most US triatomines, which may contribute to our results (Klotz et al., 2016). Little research has been focused on ectoparasites of triatomines in the US since the 1970s (Anderson, 1968). However, parasitism by mites on CD vectors may be an important factor resulting in low human infection in this region. Additional epidemiologic and entomologic investigations are recommended to examine the CD dynamics in the BBNP and Terlingua areas of Texas.
